# Effect of Facilitation of Local Maternal-and-Newborn Stakeholder Groups on Neonatal Mortality: Cluster-Randomized Controlled Trial

**DOI:** 10.1371/journal.pmed.1001445

**Published:** 2013-05-14

**Authors:** Lars Åke Persson, Nguyen T. Nga, Mats Målqvist, Dinh Thi Phuong Hoa, Leif Eriksson, Lars Wallin, Katarina Selling, Tran Q. Huy, Duong M. Duc, Tran V. Tiep, Vu Thi Thu Thuy, Uwe Ewald

**Affiliations:** 1 International Maternal and Child Health, Department of Women's and Children's Health, Uppsala University, Uppsala, Sweden; 2 Vietnam-Sweden Uong Bi General Hospital, Uong Bi, Viet Nam; 3 Hanoi School of Public Health, Hanoi, Viet Nam; 4 Department of Neurobiology, Care Sciences and Society, Division of Nursing, Karolinska Institutet, Stockholm, Sweden, and School of Health and Social Studies, Dalarna University, Falun, Sweden; 5 Ministry of Health, Hanoi, Viet Nam; 6 Provincial Health Bureau, Quang Ninh Province, Viet Nam; Columbia University Mailman School of Public Health, United States of America

## Abstract

**Background:**

Facilitation of local women's groups may reportedly reduce neonatal mortality. It is not known whether facilitation of groups composed of local health care staff and politicians can improve perinatal outcomes. We hypothesised that facilitation of local stakeholder groups would reduce neonatal mortality (primary outcome) and improve maternal, delivery, and newborn care indicators (secondary outcomes) in Quang Ninh province, Vietnam.

**Methods and Findings:**

In a cluster-randomized design 44 communes were allocated to intervention and 46 to control. Laywomen facilitated monthly meetings during 3 years in groups composed of health care staff and key persons in the communes. A problem-solving approach was employed. Births and neonatal deaths were monitored, and interviews were performed in households of neonatal deaths and of randomly selected surviving infants. A latent period before effect is expected in this type of intervention, but this timeframe was not pre-specified. Neonatal mortality rate (NMR) from July 2008 to June 2011 was 16.5/1,000 (195 deaths per 11,818 live births) in the intervention communes and 18.4/1,000 (194 per 10,559 live births) in control communes (adjusted odds ratio [OR] 0.96 [95% CI 0.73–1.25]). There was a significant downward time trend of NMR in intervention communes (*p* = 0.003) but not in control communes (*p* = 0.184). No significant difference in NMR was observed during the first two years (July 2008 to June 2010) while the third year (July 2010 to June 2011) had significantly lower NMR in intervention arm: adjusted OR 0.51 (95% CI 0.30–0.89). Women in intervention communes more frequently attended antenatal care (adjusted OR 2.27 [95% CI 1.07–4.8]).

**Conclusions:**

A randomized facilitation intervention with local stakeholder groups composed of primary care staff and local politicians working for three years with a perinatal problem-solving approach resulted in increased attendance to antenatal care and reduced neonatal mortality after a latent period.

**Trial registration:**

Current Controlled Trials ISRCTN44599712

*Please see later in the article for the Editors' Summary*

## Abstract 2

Lars Åke Persson and colleagues conduct a cluster randomised control in northern Vietnam to analyze the effect of the activity of local community-based maternal-and-newborn stakeholder groups on neonatal mortality.

*Please see later in the article for the Editors' Summary*

## Editors' Summary

**Background:**

Over the past few years, there has been enormous international effort to meet the target set by Millennium Development Goal 4 to reduce the under-five child mortality rate by two-thirds and to reduce the number of maternal deaths by three-quarters, respectively, from the 1990 level by 2015. There has been some encouraging progress and according to the latest figures from the World Health Organization, in 2011, just under 7 million children aged under 5 years died, a fall of almost 3 million from a decade ago. However, currently, 41% of all deaths among children under the age of 5 years occur around birth and the first 28 days of life (perinatal and neonatal mortality). Simple interventions can substantially reduce neonatal deaths and there have been several international, national, and local efforts to implement effective care packages to help reduce the number of neonatal deaths.

**Why Was This Study Done?:**

In order for these interventions to be most effective, it is important that the local community becomes involved. Community mobilization, especially through local women's groups, can empower women to prioritize specific interventions to help improve their own health and that of their baby. An alternative strategy might be to mobilize people who already have responsibility to promote health and welfare in society, such as primary care staff, village health workers, and elected political representatives. However, it is unclear if the activities of such stakeholder groups result in improved neonatal survival. So in this study from northern Vietnam, the researchers analyzed the effect of the activity of local maternal-and-newborn stakeholder groups on neonatal mortality.

**What Did the Researchers Do and Find?:**

Between 2008 and 2011, the researchers conducted a cluster-randomized controlled trial in 90 communes within the Quang Ninh province of northeast of Vietnam: 44 communes were allocated to intervention and 46 to the control. The local women's union facilitated recruitment to the intervention, local stakeholder groups (Maternal and Newborn Health Groups), which comprised primary care staff, village health workers, women's union representatives, and the person with responsibility for health in the commune. The groups' role was to identify and prioritize local perinatal health problems and implement actions to help overcome these problems.

Over the three-year period, the Maternal and Newborn Health Groups in the 44 intervention communes had 1,508 meetings. Every year 15–27 unique problems were identified and addressed 94–151 times. The problem-solving processes resulted in an annual number of 19–27 unique actions that were applied 297–649 times per year. The top priority problems and actions identified by these groups dealt with antenatal care attendance, post-natal visits, nutrition and rest during pregnancy, home deliveries, and breast feeding. Neonatal mortality in the intervention group did not change over the first two years but showed a significant improvement in the third year. The three leading causes of death were prematurity/low birth-weight (36%), intrapartum-related neonatal deaths (30%), and infections (15%). Stillbirth rates were 7.4 per 1,000 births in the intervention arm and 9.0 per 1,000 births in the control arm. There was one maternal death in the intervention communes and four in the control communes and there was a significant improvement in antenatal care attendance in the intervention arm. However, there were no significant differences between the intervention and control groups of other outcomes, including tetanus immunization, delivery preparedness, institutional delivery, temperature control at delivery, early initiation of breastfeeding, or home visit of a midwife during the first week after delivery.

**What Do These Findings Mean?:**

These findings suggest that local stakeholder groups comprised of primary care staff and local politicians using a problem-solving approach may help to reduce the neonatal mortality rate after three years of implementation (although the time period for an expected reduction in neonatal mortality was not specified before the trial started) and may also increase the rate of antenatal care attendance. However, the intervention had no effect on other important outcomes such as the rate of institutional delivery and breast feeding. This study used a novel approach of community-based activity that was implemented into the public sector system at low cost. A further reduction in neonatal deaths around delivery might be achieved by neonatal resuscitation training and home visits to the mother and her baby.

**Additional Information:**

Please access these Web sites via the online version of this summary at http://dx.doi.org/10.1371/journal.pmed.1001445.

- The World Health Organization provides comprehensive statistics on neonatal mortality
- The Healthy Newborn Network has information on community interventions to help reduce neonatal mortality from around the world

## Introduction

Perinatal and late neonatal mortality remains a challenge in low- and middle-income countries in contrast to the progress in reducing post-neonatal and child mortality [1]. A number of single interventions and packages has proven effective in reducing the number of stillbirths and/or neonatal deaths [2]–[4]. Coverage of such interventions is currently being monitored across the continuum of care from pregnancy to early childhood [1]. Scaling-up of efficacious single or package interventions may be difficult and result in low effectiveness [5]. Social and geographic variation in reach and quality of services may result in inequity in neonatal survival and obstacles in reaching child mortality goals [6]–[10].

These dilemmas have prompted the development of different social or systems interventions, e.g., through community mobilization by facilitation of local women's groups [3]. Such groups usually follow a problem-solving cycle from identification of their own prioritized perinatal problem to evaluation of the actions taken. Two trials in Nepal and India, in contexts with a relatively high initial level of neonatal mortality, have reported a reduction by 30%–45% [11],[12] that has been judged to be cost-effective [13]. A participatory community-based intervention in Uganda that was informed by local health data was also effective in reducing infant mortality [14]. Facilitation of community groups in Mumbai slums did not result in effects on health care or mortality [15]. An effort to scale-up a community-based intervention in Bangladesh did not result in lower neonatal mortality [16]. Studies of women's group interventions and perinatal outcomes are ongoing in African countries [17] but so far it is not known to what extent these approaches may be effective in contexts outside South Asia [18] and in settings with a medium-level neonatal mortality rate (NMR), i.e., in the range 15–30/1,000. The care providers have not been directly represented in these community mobilization approaches, which may preclude actions related to provision of services and quality of care. One may also question the sustainability of an approach with women's groups that are specifically established for this purpose. An alternative strategy may be to mobilize people, who already have responsibility to promote health and welfare in society, e.g., primary care staff, village health workers (VHWs), and elected representatives of local political or non-governmental organizations. It is not known whether facilitation of such local stakeholder groups may result in improved perinatal survival. Thus, the aim of this trial was to analyse the effect of facilitation of local maternal-and-newborn stakeholder groups on neonatal mortality in a province in northern Vietnam.

## Methods

**Figure 1:**
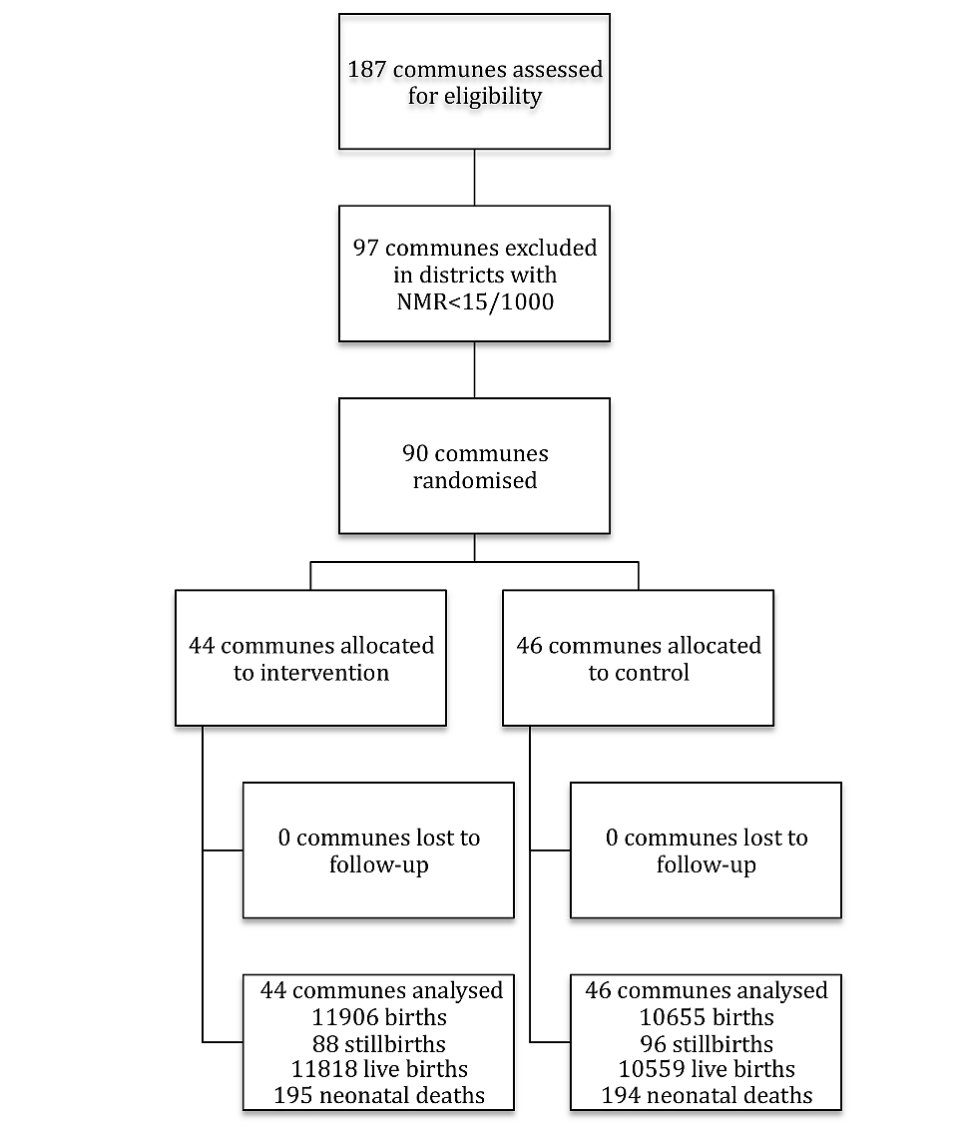
Study flow.

The study protocol of this trial has been published (Figure 1) [19].

### Study Location and Population

The trial was performed in Quang Ninh province, located in the northeast of Vietnam, which has a long coastline and borders China. There are more than 1 million inhabitants with 89% being the Kinh majority and 11% belonging to ethnic minority groups, mainly in the mountains in the western part. Vietnam, now classified as a middle-income country, had reportedly a crude birth rate in 2010 of 17/1,000, mortality before the age of 5 y of 19 per 1,000 live births, a NMR of 12 per 1,000 live births, and a maternal mortality ratio of 56 per 100,000 live births (adjusted, year 2008) [20].

A survey was performed in 2006, covering all live births and neonatal deaths in Quang Ninh province in 2005 [21]. The main aim was to map perinatal health services and analyse levels of neonatal mortality and its geographical variation and plan for this trial. Commune health centres (CHC) provided antenatal care, which was attended by three-quarters of the mothers [21]. Delivery care was offered by CHCs, or by hospitals at district, province, and regional levels. Neonatal mortality was 16/1,000 live births. A quarter of the mothers who had lost a newborn in the neonatal period had not had any contact with the health system at time of death—and this situation was more likely to be the case among mothers of ethnic minorities [22]. There was a huge variation in neonatal mortality between the districts, ranging from 10 in the urban districts along the coast to 44/1,000 live births in the mountainous inland areas.

### Procedures

**Figure 2:**
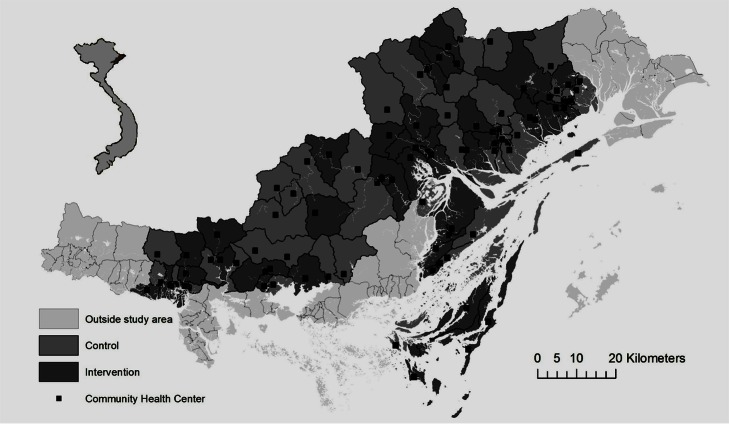
Quang Ninh province, Vietnam with study area, and randomized intervention and control communes.

The study area of this trial were 90/187 communes in the province located in districts with NMR≥15/1,000 in 2005 (Figure 2). The number of inhabitants in the different communes ranged from 1,000 to 18,000; amounting to a total of 350,000 within the study area. The rationale for excluding districts with lower mortality was the assumption, that a community-based participatory intervention potentially should be less effective in areas with a low NMR. The included districts had 6,306 births and an average NMR of 24/1,000 in 2005.

The trial had a cluster-randomized design, with the geopolitical unit *commune* constituting the cluster. An individual randomization was not possible, due to the intervention on the commune level. Each commune has a political leadership, provides services to the population including a CHC, and is a well-recognised entity in Vietnamese society. The sampling strategy was one-stage cluster sampling with probability proportional to size (PPS) of the clusters. The PPS, in this case number of births per year, was chosen to obtain similar distribution of sizes of clusters across intervention and control communes. Sampling was neither blocked nor paired. A sampling frame was established with a cumulative list of number of births in each of the communes in the 2005 survey. A random number list was used to subsequently allocate “intervention” or “control” to the list of communes, and 44 out of the 90 communes were allocated to intervention and 46 to control. The randomization was performed by one of the involved researchers at Uppsala University. The sequence was concealed until the intervention was assigned; otherwise the allocation was not masked. All mother–newborn pairs within the study area with births from July 2008 to June 2011 were eligible to be included in the trial.

The intervention consisted of facilitated work of maternal-and-child stakeholder groups on the commune level that included identification of local perinatal health problems followed by a problem-solving cycle. We hypothesised that this would improve the quality and coverage of perinatal services and after some latency lead to improved neonatal survival in comparison with control communes [19].

Primary outcome was neonatal mortality. A latent period before effect on the primary outcome is expected in this type of intervention, but this timeframe was not pre-specified. Secondary outcomes were: (a) care-seeking behaviour (in the analyses represented by attendance to antenatal care, tetanus immunisation during antenatal care, reported material and financial preparedness for delivery as part of antenatal care, and institutional delivery); (b) exclusive breast-feeding, represented by initiation of breast-feeding within 1 h; (c) temperature control at delivery (defined as the newborn placed naked and dried on mother's chest immediately after delivery in combination with the not being bathed within the first hour of life); (d) home visit by midwife during first week after delivery; and (e) perinatal health knowledge of primary health staff. The last secondary outcome is not being reported here; it will be presented in a separate publication.

Statistical power calculations were based on estimates of neonatal mortality, which was the primary outcome; NMR 24/1,000 and 6,251 live births in the study area in 2005 (on average 69 in each commune). We arbitrarily estimated the design effect to be 1.5, considering the relatively high number of clusters and the low average cluster size. A 3-y sample would allow demonstrating a significant reduction of 7/1,000 in NMR, i.e., to 17/1,000 or less, with 80% power at a 0.05 significance level. This effect size is comparable to those reported from the trials in Nepal and India [11],[12].

A steering board for the intervention was established, chaired by the hospital director of the regional Uong Bi hospital, and with members from the Women's Union (WU), the Provincial Health Bureau, the Ministry of Health, and the collaborating research institutions. The board monitored the progress of the trial and tried to motivate the local health institutions to support the efforts of the trial. The results of an interim analysis in 2010 and of all 3 y completed in 2011 were reported to the steering board. No stopping rules were applied, since we did not anticipate any negative effect of the intervention on the cluster or individual level.

We recruited lay women from the WU in the province to act as facilitators in supporting CHC staff and key commune stakeholders in improving perinatal health care practices. The WU is an organization with high national coverage working with various issues related to the situation of women in Vietnam. There is a long tradition of involvement at local and regional levels of WU in issues of welfare, particularly surrounding health care.

In a pilot study we trained two facilitators and evaluated their ability to work in this role that included participatory communication techniques and found that the facilitation strategy was feasible. Eight individuals from local WU organizations were recruited for the trial and were trained by research team members for 2 wk. Recruitment criteria included being an experienced WU member, having completed secondary school, and having children. Additionally three facilitators were recruited during the trial to replace facilitators leaving because of childbirth or new employment. The 11 facilitators (nine from the ethnic majority Kinh and two from the Tay ethnic minority group) had a mean age of 32 y at recruitment. They were paid by the project on full-time basis during the entire 3 y of intervention.

The training program of the facilitators included theoretical sessions, group discussions, role-plays, and field practice. It covered topics such as group dynamics, quality improvement methods (e.g., brainstorming and the plan-do-study-act cycle) and basic evidence-based perinatal care. A facilitation manual and a specific diary were developed to guide the work of the facilitators. Two research team members coordinated the facilitation process and acted as supervisors of the facilitators; i.e., field supervision and performing 2-d meetings with all facilitators once a month during the entire trial period.

**Figure 3:**
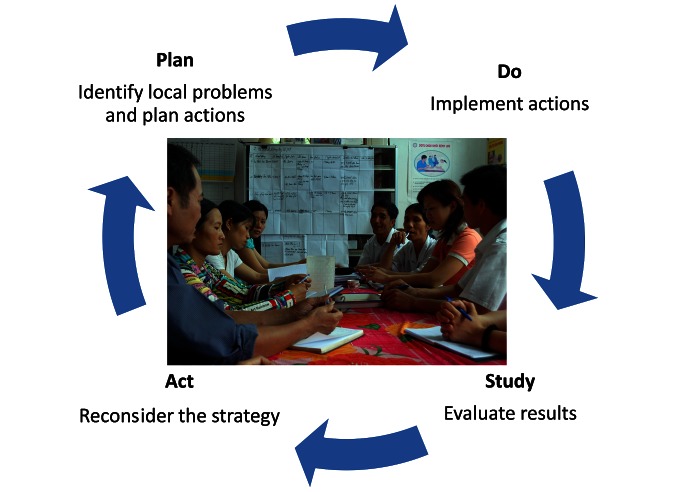
Action cycle in Maternal and Newborn Health Groups.

Maternal and Newborn Health Groups (MNHG) were constituted in each intervention commune (by directives from the Provincial Health Bureau). These groups consisted of eight members: three CHC staff (physician, midwife, nurse); one of the VHWs of the commune; one population collaborator, the chairperson, or vice chairperson of the commune (having responsibility for health in the commune); and two WU representatives (from village and commune levels). The facilitators primarily used the plan-do-study-act cycle in mobilizing the groups in identifying and priorizing local perinatal health problems and accomplishing improvement cycles that included concrete actions on prioritized problems (Figure 3). Such improvement cycles on different problems were performed continously over the intervention period in all MNHGs. We strived to keep each facilitator performing monthly meetings with the same MNHG over the 3-y intervention period. Each facilitator was responsible for five to eight MNHGs. Twenty MNHGs kept the same facilitator the whole period, 22 changed facilitator once, and two MNHGs changed facilitator twice. When appropriate, the facilitators were recommended to highlight recommendations provided by the Vietnamese National Standard and Guidelines on Reproductive Health Care Services from 2003. MNHG members were not paid and did not receive any allowances for participating in the NeoKIP project as the improvement work was assumed to be an integrated part of their normal duties. All members of the MNHGs had in their different professions important roles in the commune's health and welfare.

The facilitation process and the work of the MNHGs were monitored. Meetings, issues dealt with, and actions taken were recorded and are briefly reported in the result section. Facilitators kept diaries, and focus groups were held with facilitators and MNHG members (unpublished data).

Data collectors, who had no contact with the facilitation process, were responsible for collection of data on all births and neonatal deaths in the study area. They attended monthly meetings at CHCs, where VHWs regularly report vital events to CHC staff. They also performed monthly visits to all district hospitals and to the two provincial and regional level hospitals in the area to collect information on all births, stillbirths, maternal deaths, and neonatal deaths in the area. They kept an updated list of pregnant women, which was used to ascertain that valid information on all pregnancy outcomes was obtained. Triangulation was systematically performed of birth and neonatal death data from all included sources (records from district, provincial, and regional hospitals, records and reports from CHCs, reports provided by VHWs). Data of births and birth outcomes were carefully crosschecked between the different sources of information in order to ascertain that all births and their outcome were registered, and that no duplication of information occurred. This methodology was developed in the 2005 baseline survey and judged to provide valid information [23]. Stillbirth was defined as birth of a dead foetus after an estimated 28 wk of gestation. Live birth was defined as birth of a foetus with any sign of viability. Neonatal death was defined as death of a live birth during the first 28 d of life.

When a probable neonatal death was identified, a data collector performed a home visit to ascertain the case, and perform a verbal autopsy-based classification of cause of death [24]. A questionnaire, modified from the World Health Organization (WHO) generic verbal autopsy instrument was used in the interview that also included the mother's or other family member's story of the events prior to the neonatal death. In the training of the data collectors the importance to adhere to the pregnancy outcome definitions was stressed as well as the ability to differentiate between stillbirths and early neonatal deaths. Three paediatricians independently scrutinized the information provided and assigned a cause of death according to the WHO ICD-10 classification. In case of disagreement the cause was established in a consensus process. A questionnaire-based interview on socio-economic information and health care utilisation was also performed. Details of the cause-of-death results have been presented elsewhere [25]. Coordinates of households of all neonatal deaths were also registered with a global positioning system tool (GPS).

A 6% random sample of all registered live births, surviving the neonatal period, was continuously selected (each month) in order to represent the entire birth cohort. As for the families of the deceased newborn cases home visits were performed to families in this random sample, and interviews on socio-economic information, perinatal health care utilisation, and newborn care were performed. GPS coordinates of the households of the sample of newborns were collected. This random subsample of the population of live births will be referred to as “referents” and the newborn deaths will be referred to as “cases.”

### Analyses

Supervisors checked the data collected in the field. A second data quality control was done before computerisation, and the databases were carefully checked for completeness and consistency. Spatial information was used to produce a map of intervention and control communes and all CHCs in the area. Problems identified in the facilitated group meetings and actions taken were quantified and described. Information from first-year randomly selected birth interviews was used to describe socio-economic characteristics and basic perinatal health services utilisation in intervention and control communes. Information on births and pregnancy outcomes in the 2005 baseline survey was also analysed for intervention and control communes.

The random sample of referents was used to analyse the secondary outcomes. The cases and the sample of referents were also used in a supportive analysis of effect of the intervention on neonatal mortality outcome adjusting for baseline covariates (nested case-referent analysis).

Frequencies of births, stillbirths, live births, neonatal deaths, and maternal deaths were analysed for the entire trial period (July 2008 to June 2011) as well as for each of the three 12-mo periods (July 2008 to June 2009, July 2009 to June 2010, and July 2010 to June 2011, respectively). Stillbirth rates (per 1,000 births), perinatal mortality rates (per 1,000 births), and neonatal mortality rates (per 1,000 live births) were calculated with 95% CI.

In order to assess the homogeneity within the communes (clusters) the intraclass (or intracluster) correlation coefficient (ICC) for the binomial variable neonatal death was calculated by use of the R package “aod” [26],[27]. In “aod” (that stands for analysis of overdispersed data) point estimates of ICC for clustered binomial data are estimated using a one-way random effect model.

The effect of intervention (intervention versus control) on the primary outcome neonatal mortality (binary; deceased versus not deceased) for all live births in the study population was analysed by means of generalized linear mixed models (GLMM) using the R package “lme4” [28]. In the model intervention was included as a fixed factor, nested within the random factor commune (i.e., the clusters). Results are presented as odds ratios (OR) and 95% CIs. Analyses were performed for the entire trial period of 3 y as well as for each year separately (calendar periods defined above). Time trends with regard to neonatal mortality were analysed by including the fixed factor year (1, 2, and 3, respectively) as well as the interaction term year*intervention in the main model described above.

Further, we estimated intervention effects on neonatal mortality outcome by including adjustment for socio-economic covariates (presented in Table 1) in a nested case-referent analysis (since baseline characteristics were not available for the entire cohort). Intervention was included as a fixed factor, nested within the random factor commune (i.e., the clusters). The baseline socio-economic characteristics were included as fixed factors in the model.

**Table 1:** Baseline characteristics of mothers in intervention and control communes. Data from a random sample (398/7,033) of live births, first year of trial.

Characteristic	Intervention Communes	Control Communes
Ethnic minority household	33 (71/213)	38 (70/185)
Poor household	19 (41/213)	27 (50/185)
Mother farmer	42 (89/213)	51 (95/185)
Mother lack formal education	15 (32/213)	21 (38/185)
Mother<20 y old	8.9 (19/213)	9.2 (17/185)
First-born child	39 (84/213)	38 (71/185)

Data are percent (numerator/denominator).

The effect of intervention on the binary secondary outcomes (i.e., antenatal care, tetanus immunisation, delivery preparedness, institutional delivery, temperature control, early breastfeeding, home visits) in the previously described subsample of live births was analysed in the same manner as the analyses of the primary outcome, and time trend analyses were also performed accordingly. Also, baseline socio-economic factors (Table 1) were controlled for.

### Ethics Statement

Informed consent was sought from parents of neonates in the random sample of all live births as well as from parents of the deceased neonates at home-based interviews. The project was ethically reviewed and approved by the Ministry of Health, Hanoi, and the Regional Research Ethics Committee, Uppsala University. Before start of study the project was presented and approved at the provincial health authority level, and thereafter presented and informally approved in a series of meetings at district and commune levels.

## Results

The 90 communes in districts with NMR ≥15/1,000 in 2005 were randomly allocated to 44 intervention communes and 46 control communes (Figure 1). No communes were lost to follow-up. One intervention commune stopped the facilitation meetings after 21 mo, i.e., 15 mo before the end of trial, while all others completed the intervention. Ethnicity, economic situation, education, and utilisation of health services were similar among delivering women in randomized intervention and control communes (Table 1). Pregnancy outcomes and neonatal mortality rates in interventions and control communes had been similar in the 2005 baseline (Table 2).

**Table 2:** Neonatal mortality outcome.

Outcome	Baseline (2005)		Year 1		Year 2		Year 3		Year 1–3	
	Intervention	Control	Intervention	Control	Intervention	Control	Intervention	Control	Intervention	Control
Births	3,264	3,042	3,783	3,303	4,038	3,625	4,085	3,727	11,906	10,655
Stillbirths	26	29	23	29	37	35	28	32	88	96
Live births	3,238	3,013	3,760	3,274	4,001	3,590	4,057	3,695	11,818	10,559
Neonatal deaths	80	70	72	59	76	57	47	78	195	194
*Early (0–6 d)*	*68*	*57*	*56*	*50*	*61*	*41*	*37*	*55*	*154*	*146*
*Late (7–28 d)*	*12*	*13*	*16*	*9*	*15*	*16*	*10*	*23*	*41*	*48*
Maternal deaths	2	4	0	2	0	1	1	1	1	4
Stillbirths/1,000 births	8.0	9.5	6.1	8.8	9.2	9.7	6.9	8.6	7.4	9.0
Perinatal deaths/1,000 births	28.8	28.3	20.9	23.9	24.3	21.0	15.9	23.3	20.3	22.7
Neonatal deaths/1,000 live births (95% CI)	24.8 (20.0–30.7)	23.2 (18.4–29.3)	19.1 (15.2–24.0)	18.0 (14.0–23.2)	19.0 (15.2–23.7)	15.9 (12.3–20.5)	11.6 (8.7–15.4)	21.1 (17.0–26.3)	16.5 (14.4–19.0)	18.4 (16.0–21.1)
Adjusted OR^a^	—	—	1.08 (0.66–1.77)	1.0	1.23 (0.75–2.01)	1.0	0.51 (0.30–0.89)	1.0	0.96 (0.73–1.25)	1.0

a Adjusted for cluster-randomization, generalized linear mixed models (GLMM; binomial).

In the 44 communes allocated to the intervention the facilitated groups had 1,508 meetings (out of a possible total of 1,584 monthly meetings). On average a meeting lasted for 2 h. Every year 15–27 unique problems were identified and addressed 94–151 times. The problem-solving processes resulted in an annual number of 19–27 unique actions that were applied 297–649 times per year.

The problems most frequently identified, and the most frequent actions taken are listed in Box 1. Most problems were related to the utilisation of health services and the mother's own care of herself and her newborn. The actions most frequently dealt with communication of messages from maternal and child health service providers and counselling of mothers.

**Box 1. Most Frequently Identified Problems and Actions Taken (*n* of Times That Problem/Action Was Identified and Implemented)**

*Problems*

1. Low frequency of antenatal visits at the right time (42)
2. Low frequency of post-natal home visits (33)
3. Low awareness among pregnant women of appropriate diet, work, and rest (23)
4. High frequency of home deliveries (16)
5. Low awareness among pregnant women about appropriate breast feeding practices (14)

*Actions*

1. Communication activities (623)
2. Prepare education material and train VHWs (154)
3. Post-natal home visits (63)
4. Create lists of pregnant and newly delivered women (28)
5. Distribute leaflets (25)

There were 22,561 births registered in the study area from July 2008 to June 2011, whereof 184 resulted in stillbirths (8.2/1,000 births). Of the 22,377 live births 389 died in the neonatal period (17/1,000 live births), Table 2. Intraclass correlation coefficient (ICC) (neonatal death) was 0.0073 for the entire trial period and 0.0091 for year 3 (July 2010 to June 2011). NMR from July 2008 to June 2011 was 16.5/1,000 (195 deaths per 11,818 live births) in the intervention communes compared with 18.4/1,000 (194 per 10,559 live births) in control communes (adjusted OR 0.96 [95% CI 0.73–1.25]). There was a significant monotonic downward time trend of NMR in intervention communes but not in control communes (time trend analysis of intervention arm: year 2 versus year 1 *p* = 0.856; year 3 versus year 1 *p* = 0.007; year 3 versus year 1 and 2 *p* = 0.003. Control arm: year 2 versus year 1 *p* = 0.324; year 3 versus year 1 *p* = 0.561; year 3 versus year 1 and 2 *p* = 0.184). When evaluating the time trend in the entire material with an interaction term intervention:year the third year was also significantly different (*p* = 0.0128). No significant difference in NMR was observed during the first 2 y of the trial (July 2008 to June 2009 and July 2009 to June 2010, respectively) while the third year (July 2010 to June 2011) showed a significantly lower NMR in intervention arm; adjusted OR 0.51 (95% CI 0.30–0.89), Table 2.

An additional analysis was performed with a nested case-referent approach adjusting the neonatal mortality outcome for baseline characteristics (ethnicity, maternal education level, maternal age, and household poverty). The effect estimates showed a similar pattern as in the cohort analysis (Table S1).

Secondary outcomes represented various aspects of antenatal care, delivery care, and newborn care (Table 3). Antenatal care was significantly more common among women in intervention communes (adjusted OR 2.27, 95% CI 1.07–4.80) with a significant time trend in the intervention arm. There were no significant differences in the following secondary outcomes: tetanus immunisation as part of the antenatal services (OR 1.64, 95% CI 0.83–3.24), the presence of delivery preparedness (OR 1.33, 95% CI 0.67–2.64), institutional delivery (OR 1.88, 95% CI 0.60–5.87), temperature control at delivery (OR 1.28, 95% CI 0.50–3.25), early initiation of breastfeeding (OR 0.93, 95% CI 0.6–1.37), or home visit of a midwife during the first week after delivery (OR 1.06, 95% CI 0.51–2.20) (Table 3).

**Table 3:** Secondary outcomes, time period July 2008 to June 2011.

Outcome	Intervention	Control	OR^a^	95% CI
Antenatal care^b^	91 (596/656)	82 (482/587)	2.27	1.07–4.80
Tetanus immunisation	89 (575/646)	84 (474/566)	1.64	0.83–3.24
Delivery preparedness	83 (543/656)	78 (460/587)	1.33	0.67–2.64
Institutional delivery	91 (594/656)	87 (510/587)	1.88	0.60–5.87
Temperature control	6.2 (41/656)	5.1 (30/587)	1.28	0.50–3.25
Early breast feeding	57 (372/656)	57 (334/587)	0.93	0.64–1.37
Home visit	9.0 (59/656)	7.8 (46/587)	1.06	0.51–2.20

Analysis performed on random sample of mothers with live births (*n* = 1,243). Data are percent (numerator/denominator), adjusted OR with 95% CI.

a Generalized linear mixed models, adjusted for cluster design and socio-economic covariates (ethnic minority, lack of formal education, mother<20 y of age and poor household).

b Time trend analysis antenatal care attendance in the intervention arm with year 1 as reference, year 2 *p* = 0.066, and year 3 *p* = 0.021.

The three leading causes of death were prematurity/low birth-weight (36%), intrapartum-related neonatal deaths (30%), and infections (15%) (Table 4). During the third year of the trial a reduction in the number of deaths in intervention communes was seen in low birth weight/prematurity, intrapartum-related, as well as infectious diseases deaths.

**Table 4:** Causes of neonatal death.

Cause of death	ICD-10	Year 1		Year 2		Year 3		Year 1–3	
		Intervention	Control	Intervention	Control	Intervention	Control	Intervention	Control
Low birth weight, prematurity	P07.0-4	35	17	27	17	17	28	79	62
Intrapartum-related neonatal death	P21	25	20	25	19	11	15	62	54
Infections		5	5	15	11	7	16	27	32
*Sepsis*	P36.9	*5*	*2*	*10*	*8*	*7*	*15*	*22*	*25*
*Pneumonia*	P23	*0*	*1*	*3*	*3*	*0*	*1*	*3*	*5*
*Tetanus*	A33	*0*	*2*	*2*	*0*	*0*	*0*	*2*	*2*
Malformation	Q04–Q89	2	8	3	3	1	3	6	14
Other causes		1	6	0	2	5	9	6	17
*Hypoglycaemia*	E15	*0*	*0*	*0*	*0*	*1*	*0*	*1*	*0*
*Respiratory distress syndrome*	P22	*1*	*0*	*0*	*1*	*0*	*0*	*1*	*1*
*Umbilical haemorrhage*	P51	*0*	*2*	*0*	*0*	*0*	*3*	*0*	*5*
*Neonatal jaundice*	P58	*0*	*1*	*0*	*0*	*0*	*0*	*0*	*1*
*Intoxication*	P93	*0*	*0*	*0*	*0*	*1*	*0*	*1*	*0*
*Neglect*	P96.8	*0*	*3*	*0*	*1*	*3*	*6*	*3*	*10*
Unknown cause	P96.9	5	4	5	4	6	7	15	15
Total number of deaths		73	60	75	56	47	78	195	194

Stillbirth rates were 7.4 per 1,000 births (95% CI 5.9–8.9) in intervention arm and 9.0 per 1,000 births (95% CI 7.2–10.8) in control arm. There was one maternal death in the intervention communes and four in the control communes.

## Discussion

A randomized facilitation intervention in a Vietnamese province with local maternal and newborn stakeholder groups composed of primary care staff and local politicians, who used a problem-solving approach through monthly meetings for 3 y, resulted in reduced neonatal mortality after a latent period. The top priority problems and actions identified by these groups dealt with antenatal care attendance, post-natal visits, nutrition and rest during pregnancy, home deliveries, and breast feeding. The intervention also increased antenatal care attendance, while there were no effects on secondary outcomes around delivery and newborn care.

Baseline characteristics of mothers were similar in intervention and control communes, with the exception poor households that were slightly more common in control communes. An additional analysis with adjustment for baseline covariates using a nested case-referent approach did not change the level of effect estimates on neonatal mortality. Further, neonatal mortality rate did not differ between intervention and control communes in the 2005 baseline survey. No deviations from the protocol were observed except from one out of 44 MNHGs that ended the facilitation intervention two-thirds of the way into the trial. The facilitated intervention with MNHGs maintained a high activity with a large number of problems identified and actions taken, in spite of no extra financial benefits to the group members.

The birth and neonatal outcome data collection system was separated from the facilitation intervention activities, and it included triangulation of different data sources, careful cross-checking of data, as well as systematic control of pregnancy outcomes of the cumulative lists of pregnant women in the commune that the data collectors kept. One of the VHWs in the commune (around one out of ten) was involved in the MNHG while all VHWs were informants in the data collection system. The information on births and/or neonatal deaths provided by the VHWs who were involved in the intervention was most likely not biased, since the updated lists of pregnant women enabled a systematic enquiry on a montly basis on pregnancy outcomes, as well as a triangulation of information sources and cross-checking of data. The system for collection of outcome data was developed for the 2005 survey, and was judged to provide the best possible data on births and neonatal deaths in this setting [23].

For the study design and the facilitation intervention we were inspired by the Promoting Action on Research Implementation in Health Services model [29]. This middle-range theory highlights three major ingredients for being successful in implementing research into practice: (1) the nature of the evidence being used, (2) the quality of the context in terms of coping with change, and (3) the type of facilitation needed to ensure a successful change process. Implementation is conceived as a multifaceted intervention, rather than a more straightforward, linear process of translating knowledge from experts to the local level. In the trial we analysed the effect of facilitation of local stakeholder groups focusing maternal and neonatal health problems and actions. Excellent evidence is available for a series of effective preventive and curative activities [30],[31] that have been translated to national guidelines for the Vietnamese health system context. The intervention did not impose the guidelines on the local stakeholder groups. They were free to decide which problems to focus on and what actions to take in order to address those problems. Challenging existing practices and supporting new ways of doing things facilitated this bottom-up approach to change and development [32]. NeoKIP was a complex social intervention. It was context-specific and continuously subject to negotiation and interpretation among the involved local stakeholders. Therefore, it could not be expected that this type of intervention should result in immediate effects on neonatal mortality that might be achieved by a single or package intervention—a delay must be anticipated before any mortality reduction can be shown [2]–[4]. In the protocol of this trial a latent period was considered, but the duration of this was not pre-specified [19]. This is a limitation for the results we present, but when considering this point, it should also be noted that there was a significant downward trend in NMR in the intervention arm resulting in a reduction of neonatal mortality of public health importance in the third 12-mo period of the study (OR 0.51, 95% CI 0.30–0.89), while the control arm had no significant trend.

The intervention was strengthened by the selection of members in the MNHGs, who had professional responsibilities that related to maternal and newborn health and welfare; as midwives at health centres, as members of the powerful Women's Union, as VHWs or as local politicians. However, the NeoKIP intervention was a new approach for local stakeholders, who were not used to collaborate in this kind of group activity. This type of approach requires active and diciplined stakeholders who assess, discuss, and find ways to overcome contextual barriers that may impede the process of implementation. Thus, to succeed with this undertaking time, commitment, and perseverance were needed. MNHG members gradually identified a number of antenatal, delivery, and neonatal problems and decided on actions directed towards pregnant women and their households, the health services, or the general public. They used loudspeakers (common in Vietnamese villages) to motivate the public for antenatal care and delivery at hospitals, they trained VHWs to support an optimal utilisation of perinatal services, they produced and distributed leaflets regarding perinatal health issues, and mobilized the local community. There are several features in the causal inference framework that support the effect on neonatal mortality; the reports from the MNHGs reflect intense activities for improved perinatal health, the information on utilisation of services suggests a process of change in intervention communes that could result in improved pregnancy outcome and the change in cause-specific mortality.

Most of the problems identified by the MNHGs dealt with the demand side and less with problems of the health care providers. Changes reportedly occurred in one of the most frequent problems identified, i.e., low antenatal care attendance, where health care utilisation of women in intervention communes differed favourably from those in control communes. A major part of actions taken dealt with communication and counselling of mothers. In the Makwanpur trial in Nepal of participatory women's groups, where maternal and newborn mortality was reduced, a process evaluation indicated that women in intervention clusters attended antenatal care to a larger extent, and moved towards behaviours favourable for perinatal health [33]. In the NeoKIP trial we did not see any overall sigificant increase in institutional deliveries or care of the newborn, such as temperature control, initiation of breastfeeding, and home-visits by health personnel in the early neonatal period. A participatory approach with groups of women is maybe more likely to influence the immediate newborn care of the birthing women.

Although some reduction was noted intrapartum-related neonatal deaths or asphyxia still remained a major problem, especially in home deliveries and at district hospitals [25]. A participatory approach with local stakeholders may potentially influence quality of perinatal care, but the records of the MNHGs reveal that the main focus was on the demand side. A balanced intervention with a community participatory approach combined with efforts to improve quality of perinatal care could potentially reduce mortality even further. A further reduction of intrapartum-related neonatal deaths could be achieved by neonatal resuscitation training [34]. Home visits to the mother and her newborn child (part of Ministry of Health guidelines) and improved management of neonatal infections are also needed [35]. As part of the 2005 survey of perinatal health services and outcomes the level of knowledge of neonatal among primary care level in the study area was assessed to be low [36]. Further, lack of resources, low frequency of deliveries, lack of formal training in perinatal care, and poorly paid staff were observed barriers to keeping skills at an adequate level in the health care context [37].

This novel approach is an example of a community-based activity that was implemented into the public sector system,, where so far knowledge on effectiveness has been missing [18]. The Vietnamese health system struggles to develop and meet changing needs in a society in rapid economic growth and transition [38]. In this province the private or non-governmental sector plays a limited role in relation to maternal and neonatal health services [21]. In other provinces private care providers play a more important role, although private delivery services are limited [39]. The reports from the MNHGs tell that efforts were made to improve communication between public sector care providers and mothers, and maybe with other household members [3], which may have motivated more mothers to attend antenatal care.

The study province in northern Vietnam has a medium-level neonatal mortality and a comprehensive health system for maternal and neonatal services. Overall there were relatively few home deliveries but a great geographic and social inequity in coverage of perinatal services and level of neonatal survival [9],[10],[21]. The facilitation intervention with local stakeholder groups composed of primary care staff and local politicians working for 3 y with a perinatal problem-solving approach reduced neonatal mortality after a latent period. Incremental costs for this type of intervention are judged to be low, and mainly related to cost for the laywoman facilitator and the indirect costs of MNHGs monthly meetings (total incremental cost US$77,000 or US$6.5 per birthing woman). Quang Ninh province is relatively typical for many Vietnamese provinces, with the dominating ethnic majority in towns and most rural areas, and ethnic minority groups in the remote, mountainous areas [9]. We have shown that community-based participatory approaches to reduce neonatal mortality is not only effective in South Asian societies [3] but also in a South-East Asian society in rapid transition [40].

## Supporting Information

Alternative Language Abstract S1 Vietnamese translation of the abstract by NTN and DPH.
(DOCX)

Table S1 Neonatal mortality outcome analysed by nested case-referent approach.
(DOCX)

Text S1 Trial protocol.
(PDF)

Text S2 CONSORT statement.
(DOC)

## Abbreviations

CHC: commune health centre
MNHG: Maternal and Newborn Health Groups
NMR: neonatal mortality rate
VHW: village health worker
