# Supplementary material for: Effect of Facilitation of Local Maternal-and-Newborn Stakeholder Groups on Neonatal Mortality: Cluster-Randomized Controlled Trial
Source: PLoS Med. 2013 May 14;10(5):e1001445. doi: 10.1371/journal.pmed.1001445 (PMC3653802; doi:10.1371/journal.pmed.1001445)
Supplement: Alternative Language Abstract S1 — Vietnamese translation of the abstract by NTN and DPH. (DOCX) [file pmed.1001445.s004.docx]

***Giới thiệu*:** Tác động hỗ trợ của các nhóm phụ nữ tại địa phương trong việc giảm tử vong sơ sinh đã được báo cáo trong một số nghiên cứu. Tuy nhiên vai trò hỗ trợ của các nhóm cán bộ y tế và lãnh đạo chính quyền địa phương trong việc tăng cường chăm sóc sức khỏe chu sinh lại chưa được biết đến. Chúng tôi giả thiết rằng sự hỗ trợ của các nhóm cán bộ chủ chốt tại địa phương có thể có tác động làm giảm tử vong sơ sinh (kết quả chính) đồng thời thúc đẩy chăm sóc sức khỏe bà mẹ khi mang thai, khi đẻ và chăm sóc trẻ sơ sinh (kết quả kèm theo) ở tỉnh Quảng Ninh, Việt Nam.

***Phương pháp và kết quả:***  Thiết kế nghiên cứuchọn mẫu ngẫu nhiên cụm bao gồm 44 xã can thiệp và 46 xã đối chứng. Phụ nữ địa phương là người hỗ trợ các nhóm cán bộ y tế và cán bộ chủ chốt của chính quyền xã thực hiện các cuộc họp hàng tháng và liên tục trong 3 năm. Phương pháp tiếp cận vấn đề - giải quyết được áp dụng trong quá trình hỗ trợ. Các trẻ sinh ra và tử vong sơ sinh đều được theo dõi. Thực hiện phỏng vấn tại hộ gia đình đối với tất cả các trường hợp tử vong sơ sinh và các trẻ sống được chọn ngẫu nhiên. Chúng tôi không dự kiến trước được là phải mất bao nhiêu thời gian để việc can thiệp bắt đầu có kết quả đến giảm tử vong sơ sinh. Tỉ lệ tử vong sơ sinh (NMR) trong giai đoạn từ tháng 7/2008 đến tháng 6/2011 là 16.5/1000 (195 tử vong trong số 11818 trẻ đẻ ra sống) ở các xã can thiệp và 18.4/1000 (194 tử vong trong số 10559 trẻ đẻ ra sống) ở các xã không can thiệp (tỉ suất chênh đã điều chỉnh 0.96 [95% CI 0.73-1.25]). NMR có xu hướng giảm theo thời gian có ý nghĩa thống kê ở các xã can thiệp (p=0.003) nhưng không giảm ở các xã không can thiệp (p=0.184). Tại các xã can thiệp, NMR không có sự khác biệt có ý nghĩa thống kê trong hai năm đầu (tháng 7/2008 đến tháng 6/2010) nhưng đến năm thứ ba (từ tháng 7/2010 đến tháng 6/2011) NMR giảm một cách có ý nghĩa thống kê (tỉ suất chênh đã điều chỉnh 0.51 [95% CI 0.30-0.89]). Phụ nữ ở các xã can thiệp cũng đi khám thai nhiều hơn (tỉ suất chênh đã điều chỉnh 2.27 [95% CI 1.07-4.8]).

***Kết luận:*** Can thiệp hỗ trợ chọn mẫu ngẫu nhiên với các nhóm chủ chốt địa phương bao gồm cán bộ y tế cơ sở và cán bộ lãnh đạo địa phương làm việc trong ba năm với phương pháp tiếp cận vấn đề - giải quyết trong lĩnh vực chu sinh đã mang lại kết quả là tăng số phụ nữ đi khám thai và giảm tử vong sơ sinh sau một thời gian can thiệp.

Số đăng kí nghiên cứu Thử nghiệm***:*** Các Thử nghiệm Đối chứng hiện tại ISRCTN44599712
