# Supplementary material for: Effect of Facilitation of Local Maternal-and-Newborn Stakeholder Groups on Neonatal Mortality: Cluster-Randomized Controlled Trial
Source: PLoS Med. 2013 May 14;10(5):e1001445. doi: 10.1371/journal.pmed.1001445 (PMC3653802; doi:10.1371/journal.pmed.1001445)
Supplement: Table S1 — Neonatal mortality outcome analysed by nested case-referent approach. (DOCX) [file pmed.1001445.s001.docx]

Table S1. Neonatal mortality outcome analysed by nested case-referent approach. Neonatal death cases (n=389) and randomly selected referents of live births (n=1243)

| **Time period** | **OR^1^** | **95% CI** | **OR^2^** | **95% CI** |
| --- | --- | --- | --- | --- |
| July 2008-June 2011 | 0.99 | 0.66-1.47 | 1.06 | 0.81-1.40 |
| July 2008-June 2009 | 1.06 | 0.66-1.72 | 1.16 | 0.76-1.76 |
| July 2009-June 2010 | 1.40 | 0.82-2.38 | 1.55 | 1.01-2.37 |
| July 2010-June 2011 | 0.49 | 0.27-0.87 | 0.59 | 0.37-0.94 |

**^1^**Generalized linear mixed models, adjusted for cluster design. **^2^**Generalized linear mixed models, adjusted for cluster design and socio-economic covariates (ethnic minority, lack of formal education, mother < 20 years of age and poor household)
